# Effects of climate change and human activities on the habitat suitability of *Zeugodacus tau* (Walker, 1849) in China: perspectives from ensemble modelling and niche analysis

**DOI:** 10.3389/finsc.2026.1900075

**Published:** 2026-07-29

**Authors:** Zhangzhang He, Xinyue Chen, Tianzi Xia, Yongyue Li, Xiuqin Huang, Wenting Xiong, Yanan Gong, Xinxin Liao, Zhixiong Zhou

**Affiliations:** 1 Research Team for Crop Micronutrient Metabolism & Stress Resistance & Quality Enhancement, College of Food and Biology, Jingchu University of Technology, Jingmen, Hubei, China; 2 Institute of Entomology, College of Agriculture, Yangtze University, Jingzhou, China; 3 Key Laboratory of Sustainable Crop Production in the Middle Reaches of Yangtze River (Co-construction by Ministry and Province), Ministry of Agriculture and Rural Affairs, College of Agriculture, Yangtze University, Jingzhou, China

**Keywords:** climate change, ensemble model, human activities, niche dynamics, Zeugodacus tau

## Abstract

Climate change and human activities are significantly affecting the potential distribution pattern of pests, and the study of the spreading trend of the quarantine pest *Zeugodacus tau* (Walker, 1849) (Diptera: Tephritidae) in China is important for the development of scientific and effective prevention and control strategies. In this study, we applied ensemble modelling and niche analysis to assess changes in the suitable distribution area of *Z. tau* under different climate scenarios in China at current and in the future. The results indicated that *Z. tau* was mainly distributed in central and southern China in the current period, and climate change might promote the expansion of its suitable habitat. Meanwhile, the cumulative variance explained for both the current and future periods exceeded 92%. Additionally, Schoener’s D values ranged from 0.46 to 0.57 and Hellinger’s distance (I) values ranged from 0.62 to 0.70, suggesting moderate overlap of ecological niches of *Z. tau* under future climate scenarios. This study provides an important basis for the prediction and assessment of *Z. tau* dispersal risk in the context of climate change, as well as theoretical support for pest monitoring and control decision-making, which is of positive significance for the promotion of sustainable development of agriculture and forestry.

## 1 Introduction

*Zeugodacus tau* (Walker, 1849) (Diptera: Tephritidae) is a highly invasive and destructive fruit-boring pest (1). It is widely distributed in tropical and subtropical regions of Asia, primarily including China, India, Korea, Japan, Vietnam, Myanmar, Thailand, Laos, Bhutan, Brunei, the Philippines, Cambodia, Nepal, Singapore, Bangladesh, Malaysia, Sri Lanka and Indonesia (2, 3). As a phytophagous insect, *Z. tau* mainly feeds on melon crop, solanaceous vegetable, and a variety of tropical fruits, including *Carica papaya* L., *Momordica charantia* L., *Psidium guajava* L., *Citrullus lanatus* (Thunb.) Matsum. & Nakai, and *Capsicum annuum* L. (4, 5). It inflicts damage mainly by females piercing the fruit skin through the ovipositor and laying eggs inside the fruit. This behaviour not only causes irreversible mechanical damage to the fruit, but also provides a pathway for the invasion of pathogenic microorganisms, thus accelerating fruit decay and deterioration (6). Meanwhile, the larvae hatch in the fruit and feed directly on the flesh, resulting in the loss of commercial value of the fruit, which seriously affects the quality and yield of agricultural products (7). The average infection rate of *Z. tau* on pumpkin (Cucurbita moschata Duchesne) was reported to be as high as 73.1%, with potential economic losses to the pumpkin industry in China ranging from about 30 million to 200 million RMB per year (8). In South China, the damage of *Z. tau* has resulted in a reduction of more than 30% in the yield of melon crops, and it has now become an important quarantine target in the export trade of fruits and vegetables (9). In addition, due to its strong reproductive capacity, short developmental cycle and wide adaptability, *Z. tau* has a strong invasive ability and colonisation potential, and is able to expand rapidly over a larger geographical area (10–12). The lack of effective control measures can lead to rapid outbreaks of *Z. tau*, which can pose a serious economic threat to the fruit and vegetable industry in China.

In recent years, climate change and human activities had a profound impact on insect communities in agroecosystems (13). Global warming, the frequency of extreme weather events and changes in precipitation patterns are reshaping the geographic distribution of species, with a more pronounced impact on small-bodied insect taxa in particular, which has a rapid generational change (14, 15). Rising temperatures will not only help tropical insects such as fruit flies to improve overwintering survival, advance breeding time and extend the period of occurrence, but may also promote their expansion to higher latitudes or higher altitudes (16). At the same time, human activities (e.g., land use change, transportation, urbanization and agricultural intensification) are also indirectly accelerating the spread of invasive pests and the expansion of suitable areas through changes in habitat structure, the introduction of exotic species, and human interference with natural dispersal pathways (17, 18). The ecological niche dynamics and potential distribution patterns of invasive fruit-boring pests have become more complex under the combined drive of climate change and human disturbance (19, 20). Therefore, the systematic assessment of the future potential habitat of these pests under different climatic scenarios and human activities will not only help to identify the risk of their spread, but also help to formulate scientific and effective preventive and control measures, optimize the allocation of resources, and strengthen the quarantine supervision at entry and exit points, which is of great practical significance and application value.

Species distribution models (SDMs) are important tools for exploring the relationship between species and environmental factors and predicting potential distribution patterns, and have been widely used in the fields of pest risk assessment, early warning of biological invasions, and planning of biodiversity conservation areas (21, 22). With the development of mathematical modelling methods, ensemble modeling (EM) has received high attention for its superior predictive performance, which can significantly improve the stability and accuracy of distributional prediction by integrating the prediction results of several different algorithmic models (23). Compared with individual models, which are often limited by model structure, variable selection and data quality, the integrated approach can effectively reduce the uncertainty brought by individual models and more comprehensively reflect the characteristics of species’ responses to complex environmental gradients (24–26). In addition, the geographical distribution of species is usually determined by their own ecological niche requirements (27, 28). Therefore, SDMs based on ecological niche theory can not only be used to simulate the current potential distribution areas of species, but also further analyse the changes in ecological niches at different time scales, thus revealing the potential mechanisms of spatial remodeling of species’ habitats by climate change and human activities (29). Combined with the analysis of ecological niche overlaps, we can quantify the dynamic changes of ecological niches in different periods, which can help us understand the expansion paths and adaptation strategies of invasive species, and thus provide a scientific basis for the development of more targeted management and control measures.

In this study, based on the known occurrence records of *Z. tau*, combined with climate change and human activity data under different socio-economic scenarios (SSP1-2.6, SSP2-4.5, SSP3-7.0, and SSP5-8.5) in the current period and in the future (2050s and 2070s), we applied the Biomod2 ensemble modelling approach to construct its potentially suitable distribution area and further analyse the characteristics of its climate ecological niche changes. The objectives of our study include: (1) To compare the differences in habitat area and spatial distribution of *Z. tau*’s potentially suitable areas in the current period with and without human activities in China; (2) To predict the potential areas of colonisation risk and relative changes in *Z. tau*’s areas of potential colonisation risk in the future under climate change scenarios; and (3) To assess the degree of overlap of climatic ecological niches between the current and the future periods, and to reveal the pattern of their dynamic changes. This study contributes to an in-depth understanding of the geographic expansion potential of *Z. tau* in the context of climate change and human interference, and provides a theoretical basis and scientific support for early warning, quarantine control and green agriculture management. Meanwhile, the results of this study provide important data support and decision-making references for the risk assessment of fruit fly invasion in tropical and subtropical regions of China and the formulation of comprehensive management strategies.

## 2 Materials and methods

### 2.1 Collection of species occurrence data

In this study, occurrence data for *Z. tau* were obtained from several reliable sources to ensure comprehensiveness and accuracy. These sources include: Book information and online references (CNKI, https://kns.cnki.net/kns8s/; WOS, https://www.webofscience.com/wos; NACRC, http://museum.ioz.ac.cn), public online databases (GBIF, Global Biodiversity Information Facility, https://doi.org/10.15468/dl.x999qs; iNaturalist, https://www.inaturalist.org; CABI, Center for Agriculture and Bioscience International, https://www.cabidigitallibrary.org/doi/10.1079/cabicompendium.8741), and the data from field surveys conducted by researchers via GPS in various provinces and cities in China. By integrating these diverse data sources, we collected a total of 2127 valid distribution records worldwide.

**Figure 1:**
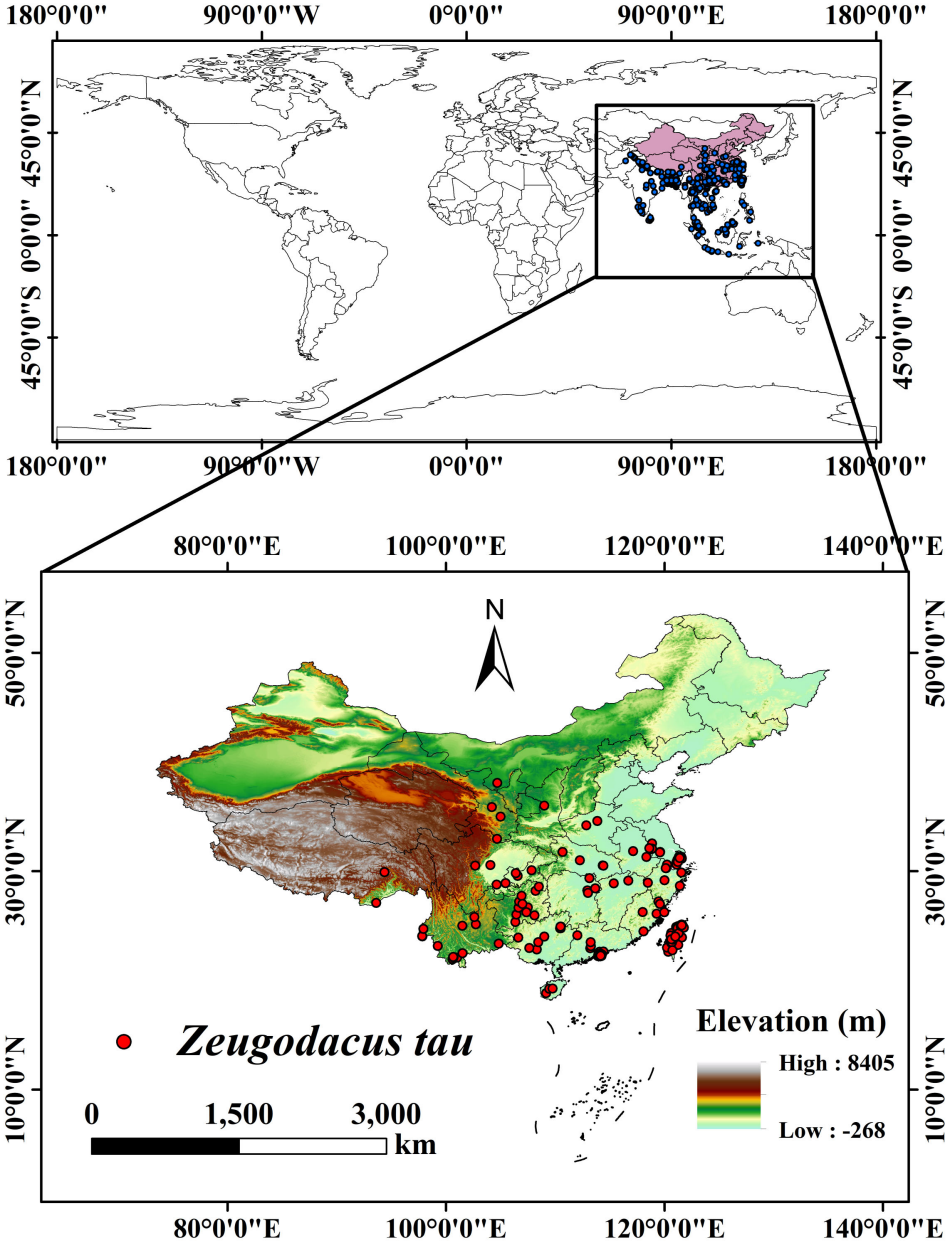
Occurrence records of *Z. tau*.

In order to reduce the risk of model overfitting caused by spatial autocorrelation, we further screened the raw distribution data. Firstly, the “raster” (version 3.6-30) and “sp” (version 2.1-4) packages of R software (version 4.4.1) were applied to extract the distribution records within the territory of China based on the administrative boundary map of China (https://www.resdc.cn/, accessed on 20 March 2025) to extract distribution records within China and remove duplicate occurrences points within each 2.5 arcmin (~4.6 km) resolution cell (30). Subsequently, the distributed data were spatially sparsified using the “spThin” package (version 0.2.0) to ensure that only one occurrence record was retained in each cell grid. This method effectively reduces the interference of spatial autocorrelation on model training and prediction performance, and improves the generalisation ability of the model (31). Ultimately, a total of 141 distribution data were screened and used for subsequent modelling analysis (**Figure 1**).

### 2.2 Collection and screening of bioclimatic variables

We downloaded 19 bioclimatic variables at a resolution of 2.5 arcmin from the WorldClim (https://worldclim.org/) climate database (version 2.1). Specifically, current climate data are based on observational records from 1970-2000. For future climate data for 2041-2060 (2050s) and 2061-2080 (2070s) the Beijing Climate Centre Climate System Model (BCC-CSM2-MR) from the Sixth International Coupled Model Intercomparison Project (CMIP6) was used, and we chose four Shared Socio-Economic Pathway (SSP) scenarios (32–34), namely the low forcing scenario (SSP1-2.6), medium forcing scenario (SSP2-4.5 and SSP3-7.0) and high forcing scenario (SSP5-8.5). In addition, we downloaded elevation data from the WorldClim database and extracted slope and aspect data using ArcGIS Map software (version 10.8.1). Solar radiation data were obtained from Helmhltz Centre for Environmental Research (https://www.ufz.de/). The normalized difference vegetation index (NDVI) and enhanced vegetation index (EVI) data were downloaded from Resource and Environmental Science Data Platform (https://www.resdc.cn). Data related to human activities are downloaded from the Socioeconomic Data and Applications Center (https://www.data4sdgs.org/resources/nasas-socioeconomic-data-and-applications-center), which includes the global human impact index (GHII), global human footprint (GHF) and global population density (PD). Finally, in order to ensure the consistency of the environmental variable data, we use the “Resample” and “Extract” tools in the ArcGIS Map software to resample and standardise all 33 environmental variables to a uniform resolution of 2.5 arcmin for subsequent modelling and analysis.

**Figure 2:**
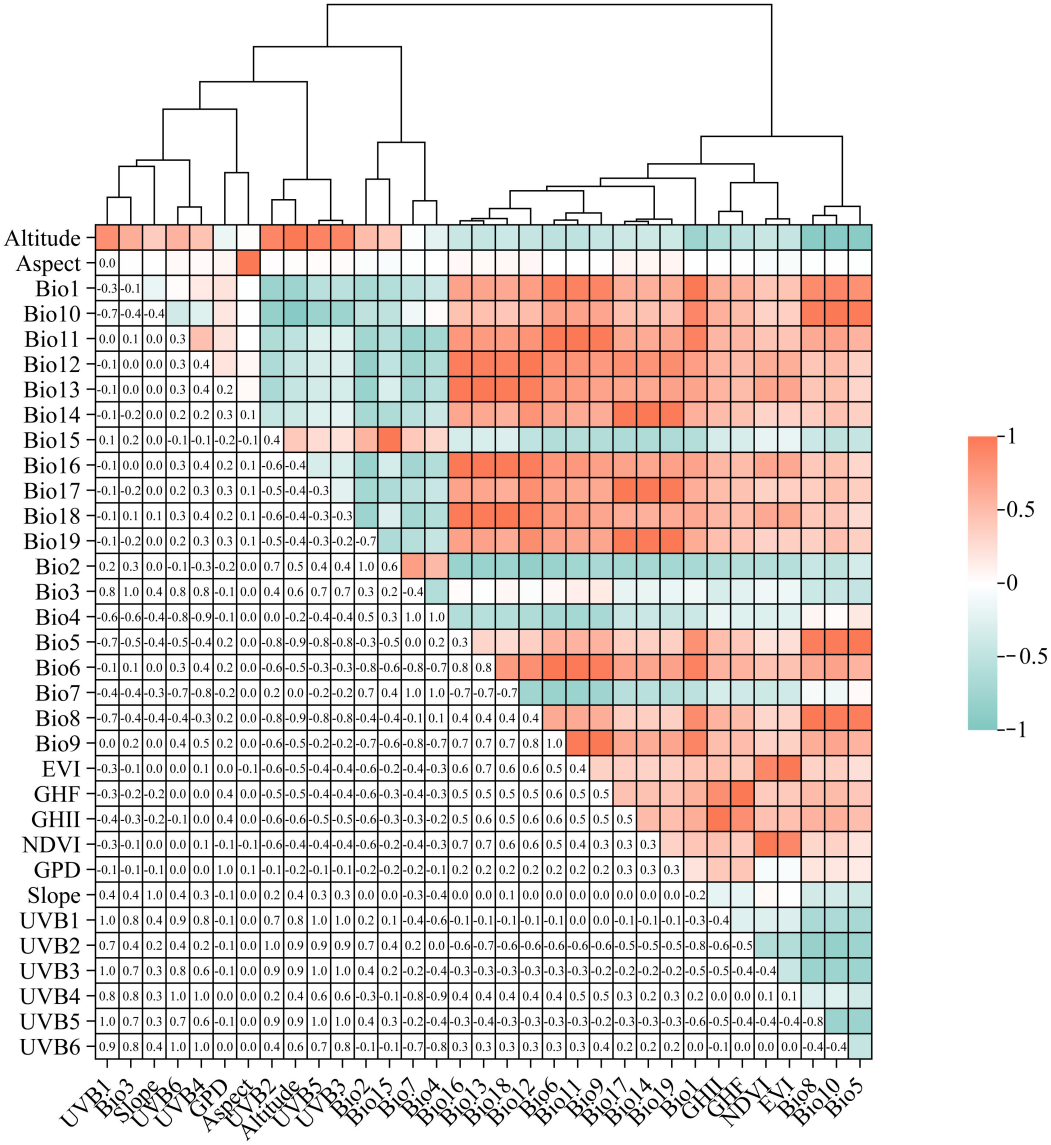
Correlation among the 33 environmental variables.

Variable selection is crucial in species distribution modeling, as high correlations between environmental factors can lead to autocorrelation and multicollinearity, thereby affecting the prediction accuracy and reliability of the model (35). To address these potential issues, we used a stepwise optimisation strategy to screen the variables. First, Pearson correlation analyses (|*r*| = 0.7) were performed on the 33 variables using the “car” package (version 3.1.2) to exclude highly correlated variables (**Figure 2**). Subsequently, the “usdm” package (version 2.1.7) was applied to calculate the variance inflation factor (VIF), and variables with VIF > 10 were gradually excluded to reduce the effect of multicollinearity (26, 31). Ultimately, 14 environmental variables that are independent of each other and make a key contribution to the model predictions were retained for modelling analysis (**Table 1**).

**Table 1:** Pearson correlation based on |*r*|=0.7 and screening environmental variables with VIF > 10.

Types	Abbreviation	Environmental variables	Operation (|*r*| < 0.7)
Bioclimate	Bio1	Annual mean temperature (°C)	Eliminate
	Bio2	Mean diurnal range (°C)	Retain
	Bio3	Isothermality	Retain
	Bio4	Temperature seasonality	Eliminate
	Bio5	Maximum temp of warmest month (°C)	Eliminate
	Bio6	Minimum temp of coldest month (°C)	Eliminate
	Bio7	Temperature annual range (°C)	Eliminate
	Bio8	Mean temp of wettest quarter (°C)	Retain
	Bio9	Mean temp of driest quarter (°C)	Eliminate
	Bio10	Mean temp of warmest quarter (°C)	Eliminate
	Bio11	Mean temp of coldest quarter (°C)	Eliminate
	Bio12	Annual precipitation (mm)	Eliminate
	Bio13	Precipitation of wettest month (mm)	Eliminate
	Bio14	Precipitation of driest month (mm)	Retain
	Bio15	Precipitation seasonality (mm)	Retain
	Bio16	Precipitation of wettest quarter (mm)	Eliminate
	Bio17	Precipitation of driest quarter (mm)	Eliminate
	Bio18	Precipitation of warmest quarter (mm)	Retain
	Bio19	Precipitation of coldest quarter (mm)	Eliminate
Topography	Altitude	Elevation (m)	Eliminate
	Aspect	Aspect	Retain
	Slope	Slope	Retain
Radiation	UVB1	Annual mean UV-B	Eliminate
	UVB2	UV-B seasonality	Eliminate
	UVB3	Mean UV-B of highest month	Retain
	UVB4	Mean UV-B of lowest month	Eliminate
	UVB5	Sum of UV-B radiation of highest quarter	Eliminate
	UVB6	Sum of UV-B radiation of lowest quarter	Eliminate
Vegetation	NDVI	Normalized difference vegetation index	Retain
	EVI	Enhanced vegetation index	Retain
Human activities	GHF	Global human footprint	Retain
	GHII	Global human influence index	Retain
	GPD	Global population density	Retain

### 2.3 Algorithms, validation and construction of ensemble models

In this study, we use the ensemble model approach to integrate the prediction results of 12 different SDMs through weighted average (Wmean) to enhance the accuracy and robustness of the model (36). The model construction and validation relied on the systematic modelling framework provided by the “Biomod2” package (version 4.2.6-2) to ensure good reliability and adaptability of the results (37). The individual models available in the “Biomod2” package include Artificial Neural Networks (ANN), Classification Tree Analysis (CTA), Flexible Discriminant Analysis (FDA), Generalized Additive Models (GAM), Generalized Boosted Models (GBM), Generalized Linear Models (GLM), Multivariate Adaptive Regression Splines (MARS), Maximum Entropy Model (MAXENT), Random Forest (RF), Random Forest with Down-sampling (RFD), Surface Range Envelope (SRE), and Extreme Gradient Boosting (XGBOOST). Each of these algorithms has its own strengths and limitations when dealing with ecological data, and combining the outputs of multiple models can help to improve the robustness of the prediction results, which can adapt to the diverse characteristics of complex ecosystems (24, 25).

In order to comprehensively assess the model performance, we used AUC (Area under the receiver operating characteristic curve) and TSS (true skill statistic) as the performance indicators to evaluate and validate the model’s accuracy. The AUC values range from 0-1, with higher values meaning that the model is better able to distinguish between the actual distribution and non-distribution areas of the species (38). The TSS value combines the sensitivity (quantification of omission error) and specificity (quantification of commission error) of the model, and takes values between -1 and 1, with values closer to 1 indicating higher model prediction accuracy (39). Therefore, combining two complementary metrics, AUC and TSS values, can be more effective in assessing the credibility and predictive performance of the model.

To ensure the reliability of the predictions, we first performed an initial evaluation of these 12 individual models, selecting only those with AUC > 0.9 and TSS > 0.8 as the basis for constructing the final ensemble model. In the modelling process, we used 80% of the species occurrence records as a training set and 20% as a test set, and randomly generated 1000 pseudo-absence points to balance the sample (40, 41). To improve the confidence of the models, modelling was repeated 10 times for each model and a 10-fold cross-validation method (k = 10) was used to reduce the model variance and enhance the robustness of the results. In addition, the tuning of the model hyperparameters relies on the automatic optimization strategy (tuned) provided by the “Biomod2” package, which further enhances the predictability and stability of the model.

### 2.4 Potential distribution area and region of *Z. tau* in the current and future periods

The effects of natural environmental variables and human activities on the geospatial distribution pattern of *Z. tau* were investigated by modeling 3 different scenarios: (1) prediction using natural environmental variables in the current period (including 2 topographic variables, 2 vegetation indices, 1 solar radiation variable, and 6 bioclimatic variables for the current period); (2) prediction using natural environmental and human activities in the current period (including 2 topographic variables, 2 vegetation indices, 1 solar radiation variable, 6 bioclimatic variables for the current period, and 3 human activity variables); and (3) prediction using natural environmental in the future period (including 2 topographic variables, 2 vegetation indices, 1 solar radiation variable, 6 bioclimatic variables for the future period, and 3 human activity variables). Scenarios (1) and (2) are prediction made based on current climate models, and scenarios (3) is a prediction based on future climate models. Scenarios (1) and (2) were used to assess anthropogenic impacts on habitat suitability of *Z. tau*, while scenarios (1) and (3) were used to explore potential impacts of climate change on habitat suitability of *Z. tau*.

We predicted the potential distribution areas of *Z. tau* using the final optimized ensemble model. The model outputs were presented in a continuous TIFF raster format, with the value of each raster representing the probability of habitat suitability (*p*) of *Z. tau* in the corresponding geographic location, which ranges from 0 to 1000, with higher values indicating a higher suitability of the raster (26, 30). To facilitate subsequent analyses, we divided the continuous suitability probability layer into binary categorized suitability maps based on the optimal specificity thresholds calculated (Human, p = 478; Non-human, p = 544). by the TSS Specifically, areas with suitability values below the threshold were judged to be unsuitable habitat, while areas above the threshold were classified as suitable. The binarization process not only improved the interpretability of the prediction results, but also helped to visualise the spatial distribution of suitable habitats of *Z. tau* under different climate scenarios in the present and future. In addition, we counted the number of rasters in the predicted suitable and unsuitable categories and further calculated the actual area of each category to quantitatively assess trends in the potential distribution range of *Z. tau*.

### 2.5 Relative changes in suitable distribution areas for *Z. tau* in future periods

In order to investigate the trend of the suitable distribution area of *Z. tau* under different climate scenarios in the future, we used the “BIOMOD_RangeSize” function in the “Biomod2” package to evaluate the relative changes of its potential distribution area (26). The function classifies the types of changes in species suitability areas into four categories: “Expansion” (species range increase), “No occupancy” (no distribution of the species under the new scenario), “No change” (species range remains unchanged), and “Contraction” (species range decreases). By comparing current and future projections, it is possible to quantitatively reveal the potential dynamics of species distribution patterns in the context of climate change.

### 2.6 Ecological niche analyses of species suitable areas under climate change scenarios

In order to assess the characteristics of future ecological niche changes in *Z. tau* under different climate scenarios, we firstly extracted “presence” and “absence” areas from the binarised maps and the corresponding values of bioclimatic variables (Bio2, Bio3, and Bio18) using ArcGIS Map. Subsequently, PCA, Schoener’s D-index and Hellinger’s I-index were calculated using the “ecospat” package (version 4.0.0) to quantitatively assess the degree of ecological niche overlap between *Z. tau* under current and future climate scenarios (42). Principal component analysis (PCA) was used to reveal the distribution patterns and trends of *Z. tau* in multidimensional climate space by compressing multiple environmental variables into two principal components. In contrast, Schoener’s D index and Hellinger’s I index measure the degree of overlap of species’ ecological niches, with values ranging from 0 to 1, where 0 indicates that the ecological niches do not overlap at all and 1 indicates complete overlap. These analyses can help reveal the stability and potential migration capacity of species ecological niches in the context of climate change, and thus provide insights into species adaptation and expansion.

## 3 Results

### 3.1 Evaluation and accuracy of ensemble model

**Figure 3:**
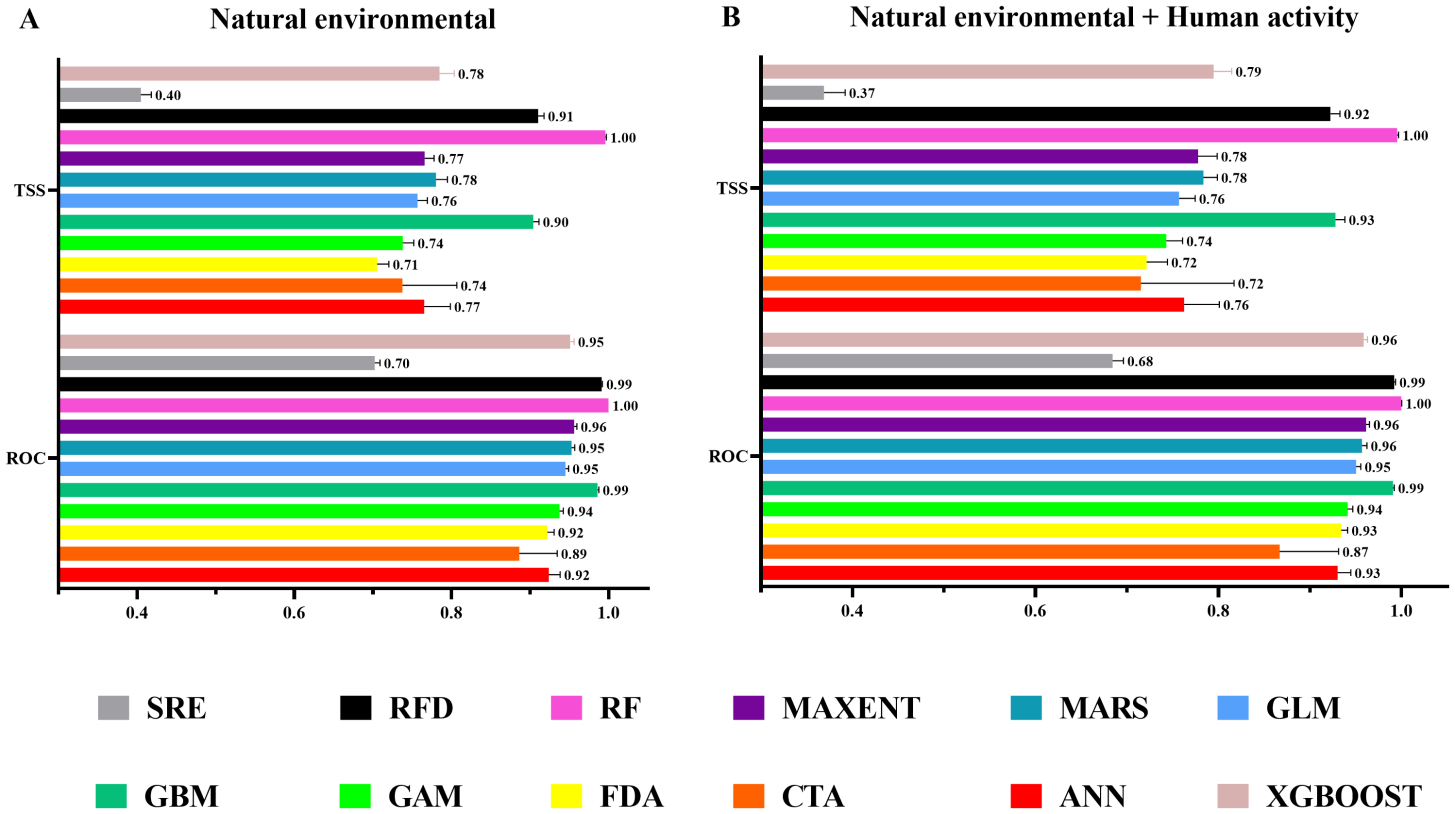
Accuracy evaluation of twelve single models. **(A)** natural environmental; **(B)** natural environmental + human activity.

In this study, we assessed the predictive performance of 12 individual models by calculating AUC and TSS values for 10 replications of each model (**Figure 3**). It is noteworthy that the combination of base models (ANN, FDA, GAM, GBM, GLM, MARS, MAXENT, RF, RFD and XGBOOST) used in the final construction of the ensemble models remained consistent, both with and without the anthropogenic variables, reflecting the strong stability and reliability of each of the models across the modelling scenarios. Specifically, in the scenario with natural environmental factors, the ensemble model had an AUC value of 0.96, a TSS value of 0.80, and Specificity and Sensitivity values of 0.92 and 0.88, respectively. When human activity variables were included in the modelling analysis, the ensemble model had an AUC value of 0.97, a TSS value was 0.81, and Specificity and Sensitivity values were 0.91 and 0.90, respectively (**Table 2**). This indicates that the two ensemble models we constructed have high accuracy and stability in predicting the potential geographic distribution range of *Z. tau*, which can provide an important reference basis for pest control strategies.

**Table 2:** Accuracy evaluation of ensemble model.

Shared socioeconomic pathways	Sensitivity	Specificity	TSS	AUC
Natural environmental	0.88	0.92	0.80	0.96
Natural environmental + Human activity	0.90	0.91	0.81	0.97

### 3.2 Predicting suitable habitat areas for *Z. tau* in the current and future periods

**Figure 4:**
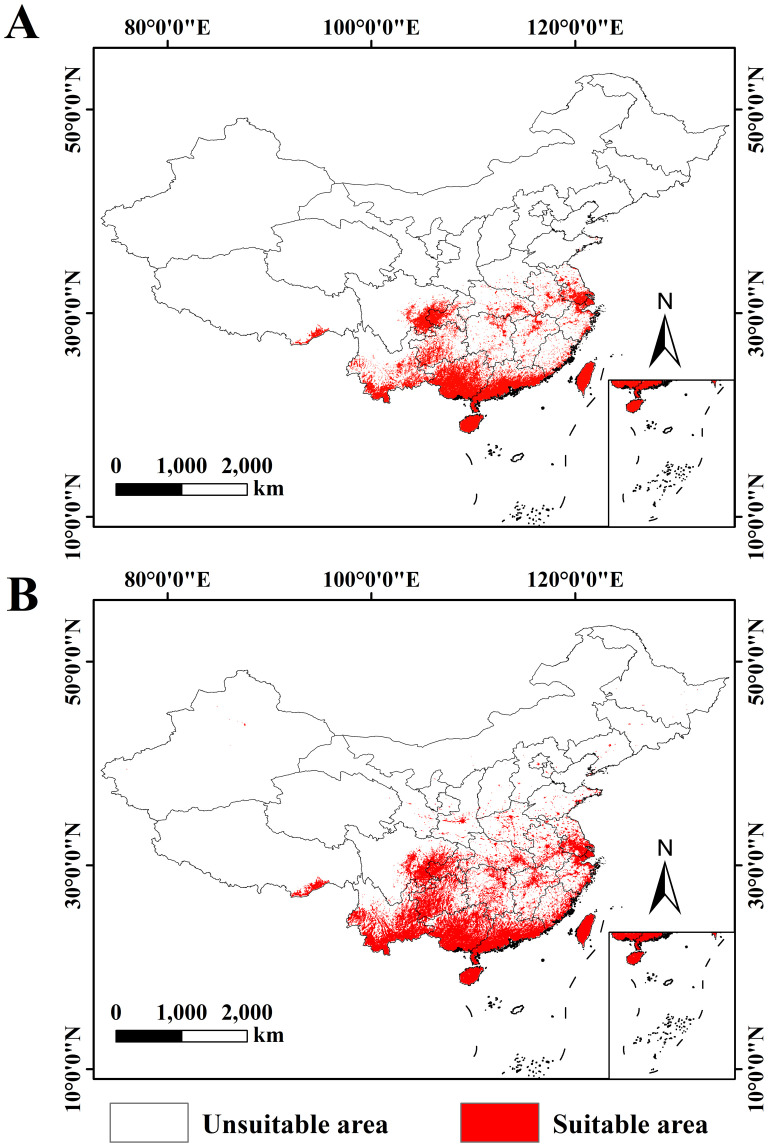
Suitable habitats for *Z. tau* under current climatic conditions. **(A)** natural environmental; **(B)** natural environmental + human activity.

The area and region of suitable distribution of *Z. tau* in the current and future periods were predicted using an ensemble model. The results indicate that *Z. tau* is mainly distributed in south and central China, including Jiangsu, Zhejiang, Anhui, Fujian, Jiangxi, Henan, Hubei, Hunan, Guangdong, Guangxi, Hainan, Chongqing, Sichuan, Guizhou, Yunnan, Hong Kong, Macao and Taiwan (**Figure 4**). Under the current natural environmental conditions, the suitable area of *Z. tau* is 64.25×10^4^ km^2^. With the addition of anthropogenic factors, the suitable area of *Z. tau* was 97.06×10^4^ km^2^ (**Table 3**). Moreover, the inclusion of human activities resulted in an increase of 32.81 × 10^4^ km^2^ in the total suitable habitat area of *Z. tau* compared to the natural environmental conditions, which suggests that human activities may have provided it with more breeding and habitat resources, thus contributing to the expansion of its geographical distribution.

**Table 3:** Predicted suitable areas for *Z. tau* under current and future climatic conditions.

Shared socioeconomic pathways	Predicted area (× 10^4^ km^2^)		Comparison with current environmental variables distribution (%)	
	Unsuitable area	Suitable area	Unsuitable area	Suitable area
Current natural environment	897.59	64.25	–	–
Current natural environment + human activity	864.77	97.06	-0.04	12.46
Future-SSP1-2.6 2041–2060	888.17	73.66	-0.01	12.82
Future-SSP1-2.6 2061–2080	876.76	85.07	-0.02	12.65
Future-SSP2-4.5 2041–2060	869.71	92.12	-0.03	12.54
Future-SSP2-4.5 2061–2080	877.42	84.41	-0.02	12.66
Future-SSP3-7.0 2041–2060	880.52	81.31	-0.02	12.71
Future-SSP3-7.0 2061–2080	884.27	77.56	-0.01	12.76
Future-SSP5-8.5 2041–2060	871.15	90.69	-0.03	12.56
Future-SSP5-8.5 2061–2080	861.79	100.04	-0.04	12.41

**Figure 5:**
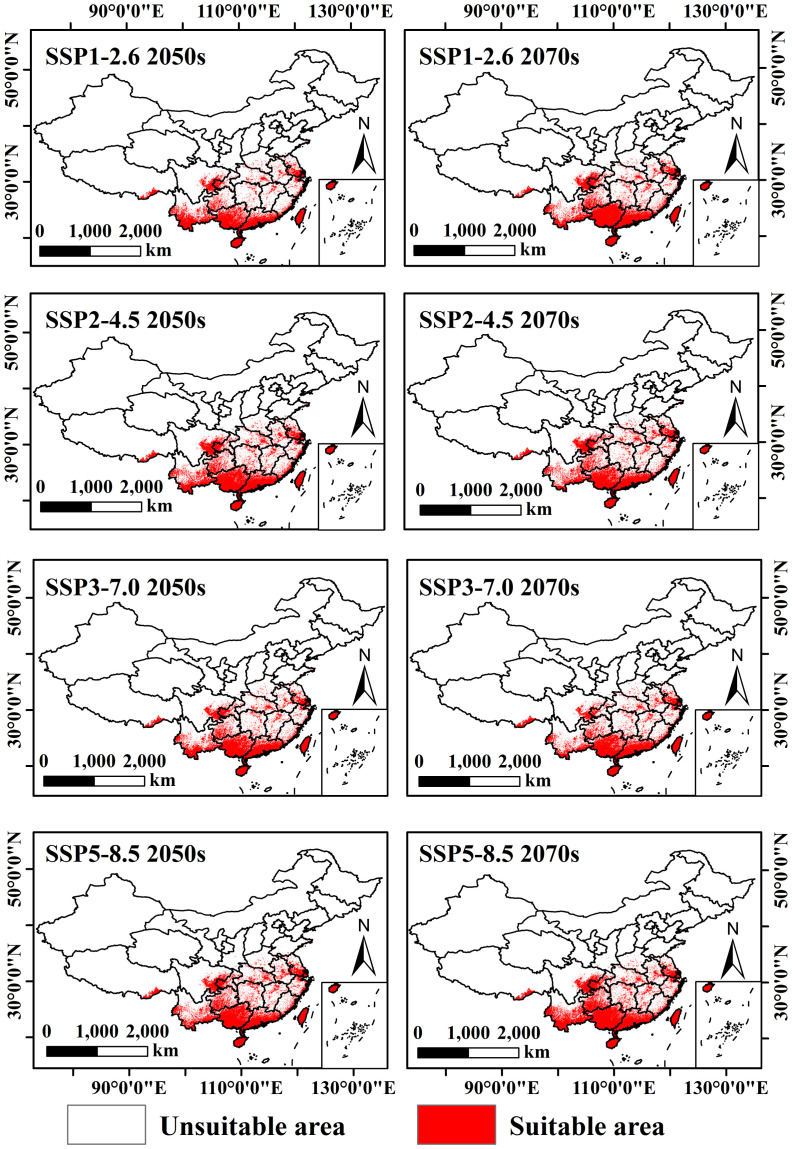
Suitable habitats for *Z. tau* under future climatic conditions.

The potential distribution area of *Z. tau* showed an increasing trend driven by climate change, suggesting that future climate warming may provide suitable conditions for its further expansion in China (**Figure 5**). Specifically, the potential area of suitable habitat for *Z. tau* under future natural environmental conditions ranged from 73.66 to 100.04 × 10^4^ km^2^. Among them, SSP5-8.5-2070s had the largest area of suitable habitat, followed by SSP2-4.5-2050s, while SSP1-2.6-2050s had the smallest area of suitable habitat. In addition, under the SSP2-4.5 and SSP3-7.0 scenarios, the area of suitable habitat for *Z. tau* tends to increase from the current period to 2050 and gradually decreases in the 2070s (**Table 3**). In contrast, under the SSP1-2.6 and SSP5-8.5 scenarios, the area of suitable habitat for *Z. tau* continues to increase over time.

### 3.3 Relative changes in suitable habitat for *Z. tau* under climate change scenarios

**Figure 6:**
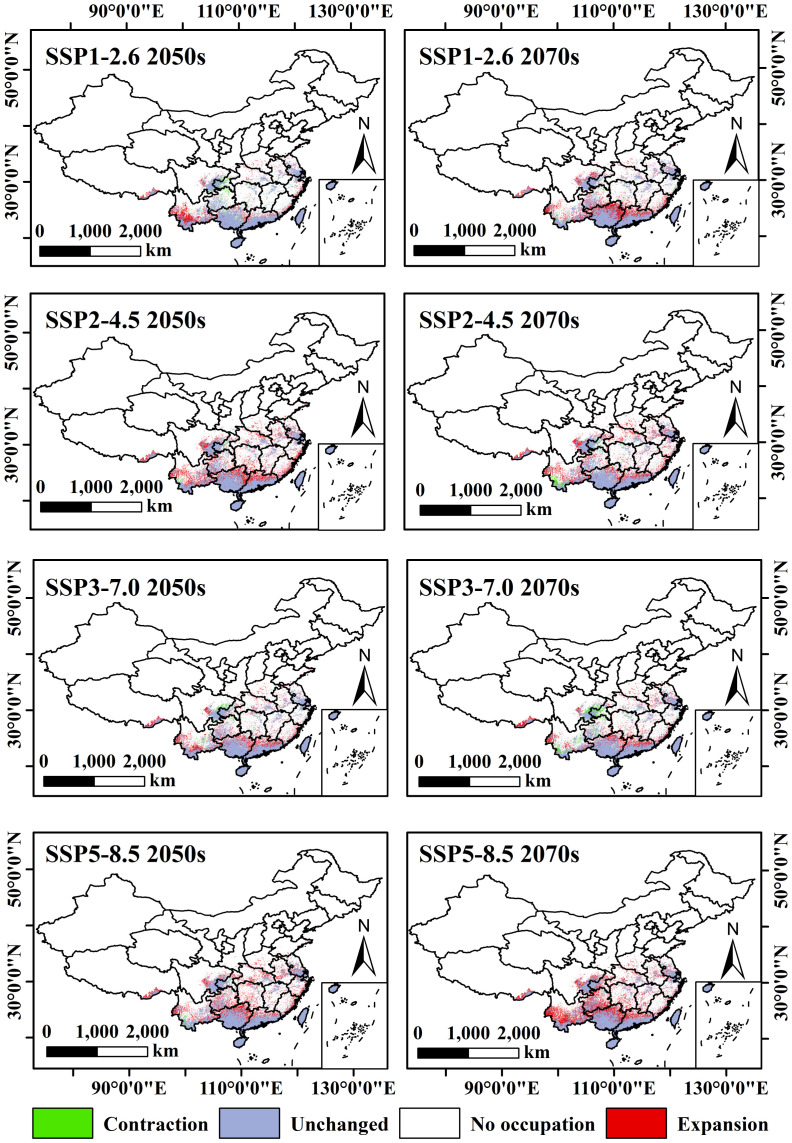
Areas of relative change for *Z. tau* under future climate scenarios.

Under future climate scenarios, *Z. tau* habitat suitability shows different relative trends in geospatial patterns (**Figure 6**). Specifically, the contracted areas of suitable habitat were mainly distributed in southern China, including Yunnan, Chongqing, Guizhou, Hubei, Hunan, Guangxi, Jiangxi, Guangdong, Fujian, Anhui, and Zhejiang, with contracted areas ranged from 0.13 to 4.37 × 10^4^ km^2^ (**Table 4**). Among them, the SSP3-7.0-2070s scenario had the largest contraction area, followed by SSP1-2.6-2050s, while SSP5-8.5-2070s had the smallest contraction area. In contrast, *Z. tau* expanded in Yunnan, Chongqing, Sichuan, Guizhou, Xizang, Hubei, Hunan, Henan, Guangxi, Jiangsu, Jiangxi, Guangdong, Fujian, Anhui, and Zhejiang, with expansion areas ranging from 13.12 to 35.93 × 10^4^ km^2^ (**Table 4**). Among them, the SSP5-8.5-2070s scenario has the largest contraction area, followed by SSP2-4.5-2050s, while SSP1-2.6-2070s has the smallest expansion area. It is noteworthy that the expansion area of *Z. tau* suitable areas was generally larger than the contraction area under the future scenarios, and the expansion trend was more pronounced under the high-emission scenario, suggesting that climate warming may provide more new suitable habitats for *Z. tau*.

**Table 4:** Area of relative change of *Z. tau* under different future climate scenarios.

Shared socioeconomic pathways	Predicted area (× 10^4^ km^2^)			
	Contraction	Unchanged	No occupancy	Expansion
Future-SSP1.0-2.6 2041–2060	3.70	60.55	884.47	13.12
Future-SSP1.0-2.6 2061–2080	1.12	63.13	875.65	21.94
Future-SSP2.0-4.5 2041–2060	0.72	63.53	869.00	28.59
Future-SSP2.0-4.5 2061–2080	2.15	62.10	875.27	22.32
Future-SSP3.0-7.0 2041–2060	1.91	62.33	878.61	18.98
Future-SSP3.0-7.0 2061–2080	4.37	59.88	879.91	17.68
Future-SSP5.0-8.5 2041–2060	0.72	63.53	870.43	27.16
Future-SSP5.0-8.5 2061–2080	0.13	64.11	861.66	35.93

### 3.4 Contribution of key environmental variables and niche analysis

**Figure 7:**
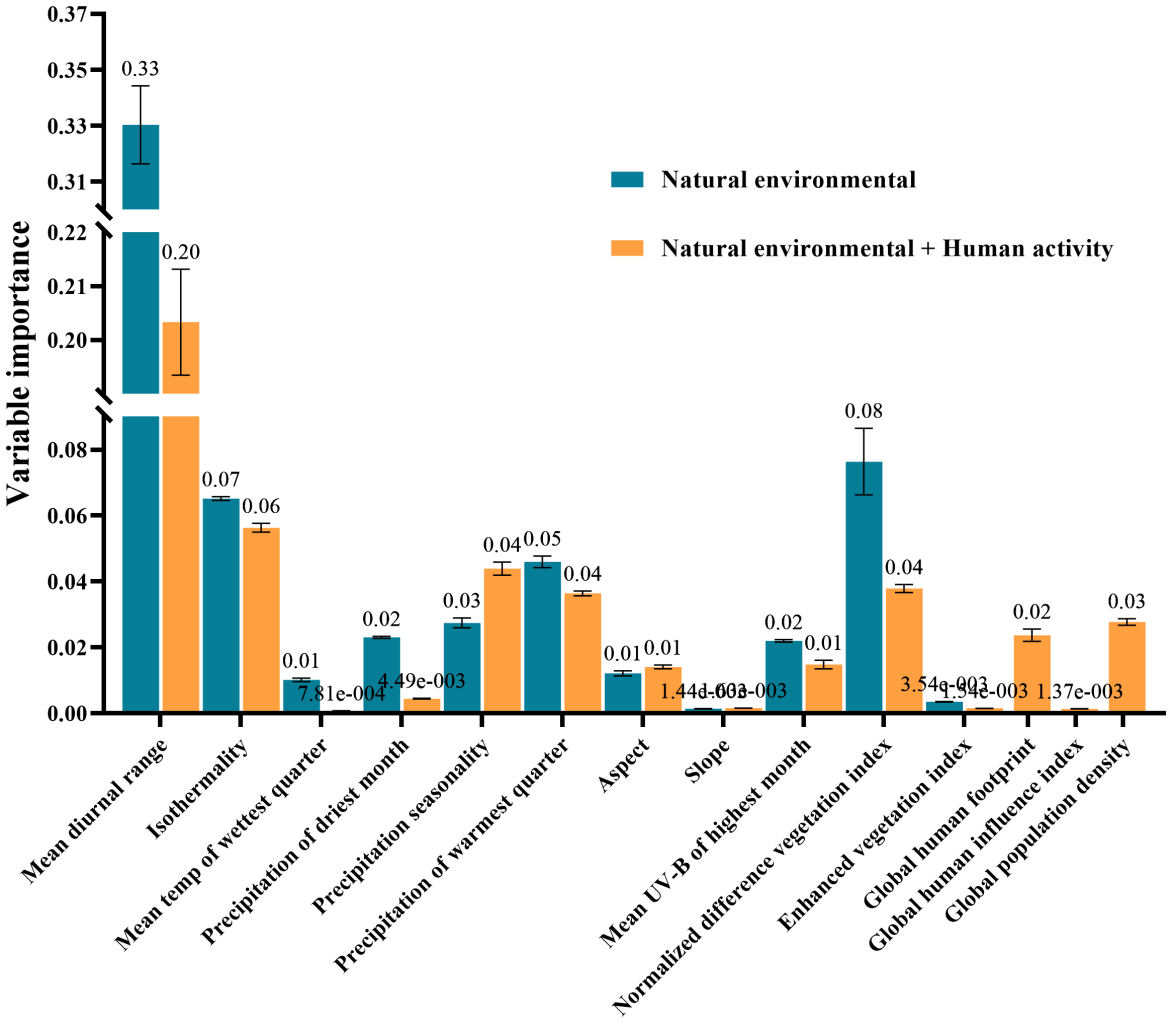
Contribution of environmental factors.

In assessing the importance of environmental factors in different scenarios, it was found that natural environmental factors played a major role in the prediction model (**Figure 7**). Specifically, under the condition of the natural environmental factor, mean diurnal range (Bio2, 0.40) has the highest contribution, followed by enhanced vegetation index (EVI, 0.078) and precipitation of warmest quarter (Bio18, 0.076). Although the other natural environmental variables had relatively low contribution values (all less than 0.06), they also played a role in predicting suitable habitat for *Z. tau*. In contrast, under the combined effect of the natural environment and human activities, despite the diminishing importance of each natural environmental variable, mean diurnal range (Bio2, 0.21) remained the dominant contributor, followed by precipitation seasonality (Bio15, 0.05) and enhanced vegetation index (EVI, 0.04). This suggests that the natural environment is always the main determinant driving the potential distribution of *Z. tau* with and without anthropogenic factors, reflecting the high sensitivity of *Z. tau* to climatic conditions. In addition, anthropogenic related variables also contributed to the model results, for example, global human footprint and global population density contributed 0.03 and 0.02 respectively. This suggests that the inclusion of anthropogenic factors not only enhances the model’s responsiveness to anthropogenic disturbances, but also further confirms that the spatial distribution pattern of *Z. tau* is shaped by both climate change and human activities.

**Figure 8:**
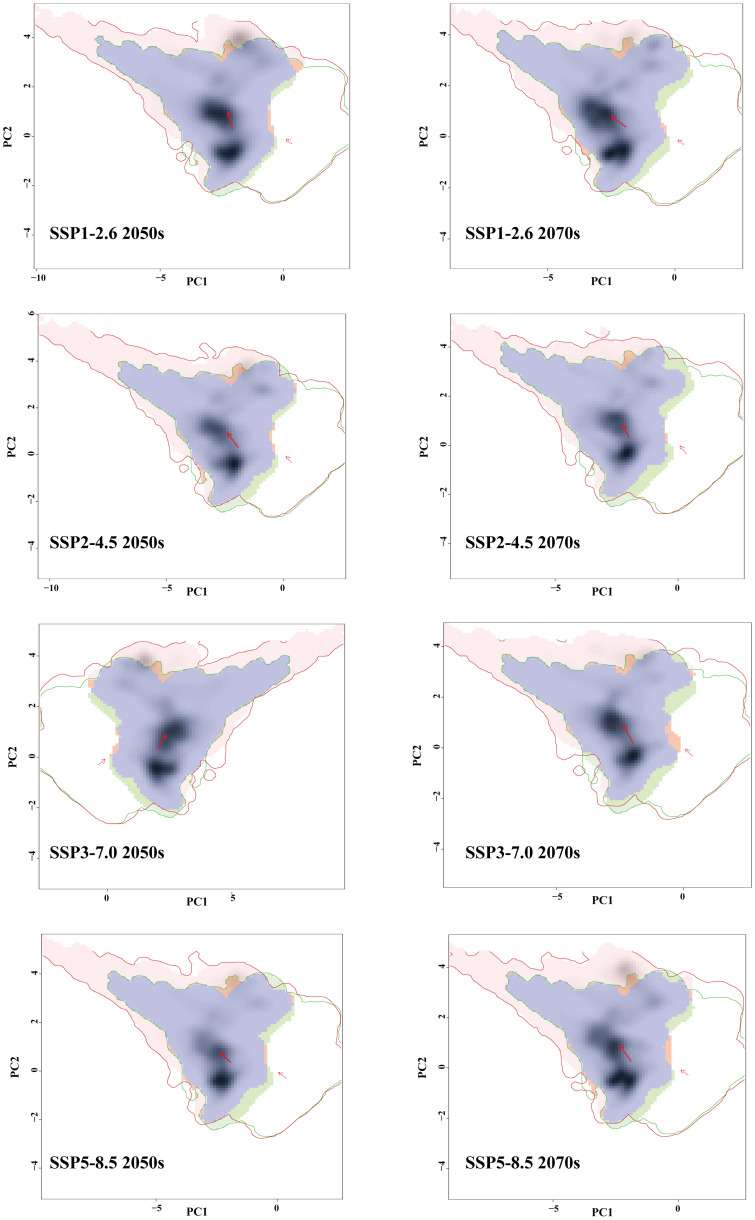
Ecological niche dynamics of *Z. tau* under different future scenarios.

The ecological niche of *Z. tau* showed moderate stability under different future climate scenarios (**Figure 8**). The results of Principal Component Analysis (PCA-env) showed that the explanatory rate of PC1 ranged from 57.20% to 58.84%, PC2 ranged from 33.96% to 35.35%, and the cumulative PC was more than 92%, which indicates that the distribution of the three main bioclimatic factors (Bio2, Bio3, and Bio18) is more concentrated in the principal component space (**Table 5**). Meanwhile, the niche overlap of *Z. tau* was relatively stable. Specifically, Schoener’s D values ranged from 0.46 to 0.57, while Hellinger’s distance index (I) ranged from 0.62 to 0.70. Among them, *Z. tau* had the highest ecological niche overlap in the SSP2-4.5-2070s scenario (D = 0.57, I = 0.70) and relatively low ecological niche overlap in the SSP2-4.5-2050s scenario (D = 0.46, I = 0.62). This suggests that climate change will have a relatively small impact on *Z. tau* habitat suitability and that *Z. tau* may still have strong ecological niche adaptations under future climate conditions.

**Table 5:** Niche comparisons and variation in principle components PC1 and PC2 between current and future projected distribution range of *Z. tau*.

Shared socioeconomic pathways	PC1 (%)	PC2 (%)	Cumulative PC (%)	Schoener’s (D)	Hellinger’s (I)
Future-SSP1-2.6 2041-2060	58.23	34.68	92.91	0.46	0.66
Future-SSP1-2.6 2061-2080	58.84	33.96	92.80	0.53	0.68
Future-SSP2-4.5 2041-2060	58.77	34.23	93.00	0.46	0.62
Future-SSP2-4.5 2061-2080	58.51	34.45	92.96	0.57	0.70
Future-SSP3-7.0 2041-2060	58.64	34.31	92.95	0.51	0.68
Future-SSP3-7.0 2061-2080	57.20	35.35	92.55	0.48	0.63
Future-SSP5-8.5 2041-2060	58.67	34.11	92.78	0.50	0.64
Future-SSP5-8.5 2061-2080	57.71	34.67	92.38	0.48	0.64

## 4 Discussion

In the context of global climate change and intensifying human activities, the spread and expansion of quarantine pests is being characterised by increasingly complex dynamics (1, 3, 20). The potential distribution range of *Z. tau*, an important quarantine fruit-boring pest, is being driven by multiple environmental factors (11, 43, 44). In order to effectively address this challenge, this study comprehensively assessed the geographic distribution pattern of *Z. tau* and its dynamics under the combined effects of the natural environment and human activities by constructing an optimised ensemble model and climate ecological niche analysis. The results of the study not only reveal the role of climate change and human interference in driving the expansion areas of the species, but also provide theoretical support for the identification of high-risk areas and the formulation of prospective prevention and control strategies, which is of great significance for strengthening the prediction, warning and management of major quarantine pests in China.

The ensemble model not only showed excellent predictive ability under different combinations of variables, but also revealed the dispersal mechanism driven by both natural environment and human activities (25). The ensemble model constructed in this study showed high robustness and predictive performance under different modelling scenarios, indicating its adaptability and practicality in assessing the potential distribution of *Z. tau*. It is noteworthy that the single model combinations selected for the final ensemble models with and without anthropogenic factors are consistent (including ANN, FDA, GAM, GBM, GLM, MARS, MAXENT, RF, RFD, and XGBOOST), reflecting the stable performance of these models when confronted with complex ecological environments and their applicability to a wide range of environmental variables. Specifically, in the model for the natural environmental factor, the average AUC value was 0.96, the TSS value was 0.80, and Specificity and Sensitivity were 0.92 and 0.88, respectively, showing the model’s ability to discriminate well between suitable and unsuitable habitats. However, when human activities were included, the model performance was further improved, with an average AUC of 0.97, TSS of 0.81, Specificity and Sensitivity of 0.91 and 0.90, respectively, which indicated that human activities could help to enhance the discriminative ability of the model, especially in the delineation of the boundary area, so that the prediction results could be more close to the actual distribution pattern. This change may stem from profound anthropogenic interventions in environmental conditions, such as agricultural practices, urbanization and infrastructure development that not only alter surface ecological patterns, but also provide *Z. tau* with richer food resources and habitats, such as warmer climates under the urban heat island effect and irrigation facilities in orchards (45, 46). Therefore, the inclusion of human activities in the modelling variables can help to reflect the driving mechanism of suitable habitat formation more comprehensively. The results of this study provide a scientific basis for accurately assessing the potential spread risk of *Z. tau*, as well as data support and theoretical references for the development of more targeted prevention and control strategies.

As a highly environmentally sensitive pest, an increase in temperature may accelerate the reproduction rate of *Z. tau*, which in turn may contribute to the expansion of its habitat range (2, 43). This study indicated that the potential distribution of *Z. tau* in China is mainly concentrated in the southern and central regions. Moreover, the area of its suitable habitat area increased by 32.81 × 10^4^ km² after the inclusion of anthropogenic activities compared with the scenario of the natural environment factor, suggesting that anthropogenic activities provided *Z. tau* with more habitat and breeding resources, which in turn promoted the expansion of its suitable habitat. This phenomenon may be related to the profound changes in the pattern of the natural environment caused by human activities, such as agricultural expansion, crop cultivation, urban greening, and infrastructure development, which not only enriched the types of resources in the local area, but also may have created microclimatic conditions favourable to the survival of the species (47, 48). Meanwhile, the urban heat island effect has led to an increase in local temperatures, and although the extreme high temperatures may pressure the survival of *Z. tau*, it also reflects that the species is able to effectively utilise the new habitats modified by human beings, and shows strong ecological adaptive capacity and dispersal potential (49). It is noteworthy that under different climate scenarios in the future, the expansion of suitable habitats for *Z. tau* is significantly larger than its contraction, a trend that may be closely related to the environmental changes induced by global warming. The increase in temperature may provide more favourable conditions for *Z. tau* to survive and reproduce, which will not only help *Z. tau* to shorten its generation cycle and accelerate its reproduction rate, but also gradually transform the unsuitable areas into suitable habitats, thus promoting the expansion of its distribution to higher latitudes and wider areas. Furthermore, changes in precipitation patterns may also have a significant impact on the distribution of *Z. tau*. On the one hand, excessive precipitation may lead to high humidity in suitable habitats, affecting their normal habitat (2, 43). On the other hand, insufficient precipitation may result in a lack of water, which may affect plant growth, thus reducing the resources on which *Z. tau* depends and indirectly limiting its expansion (1, 3, 44). In summary, human activities and climate change together contribute to the expansion of suitable habitats for *Z. tau*. Human activities have created new habitat opportunities for *Z. tau*, mainly by altering surface environmental structure and resource patterns, while climate change has radically expanded its potential range by improving bioclimatic conditions. These results not only reveal the main environmental drivers of the potential spread of *Z. tau*, but also provide a scientific basis for the development of more targeted prevention and control strategies, suggesting that the overlapping effects of natural environmental changes and human activities should be considered in future pest management practices.

Understanding the stability of species ecological niches in the context of climate change is the theoretical basis and important prerequisite for assessing their future dispersal potentials and environmental adaptation risks (35, 50). In this study, the first two axes of principal component analysis (PC1 and PC2) cumulatively explained more than 92% of the variance in environmental variables under different climate scenarios, which not only ensured the robustness of the comparative niche analysis, but also helped to identify the major environmental gradients and ecological constraints in the potential habitats of *Z. tau*. Meanwhile, further quantification of the magnitude of niche change by the ecological niche overlap metrics (Schoener’s D and Hellinger’s I) revealed that the D (0.46-0.57) and I (0.62-0.70) values were generally maintained at a moderately high level under the future scenarios, suggesting that the niche of *Z. tau* has a certain degree of stability and persistence under the future scenarios. This stability reflects its niche conservatism, i.e., the species tends to maintain the niche characteristics formed during the evolutionary process, and prefers similar ecological space even when the environmental conditions change (51–53). This not only suggests that *Z. tau* has a strong ecological adaptive capacity in the context of climate change, but also suggests that it may be able to expand to new areas in the future at a relatively small ecological cost. In particular, under the trend of increasing temperatures, the species may continue to maintain its niche requirements unchanged while migrating to higher latitudes or altitudes to fill in new suitable habitats. The strong ecological adaptability of the species may result from a combination of several biological traits, such as good tolerance to high environmental temperatures, adaptability to diverse host plants, high reproductive efficiency, and the ability to respond quickly to human-disturbed environments (2, 3). These traits not only enhance their ability to survive and reproduce in different climatic contexts, but also provide an intrinsic driving mechanism for their niche structure to remain stable in future environments.

As a fruit fly with high adaptability and expansion potential, *Z. tau* will expand to more areas under the influence of climate change and human activities. The spreading process is not only driven by bioclimatic factors but also facilitated by human activities, especially agricultural cultivation, urbanization processes and globalised trade (9, 11, 50). Therefore, the development of effective management and control measures should take into full consideration the climatic characteristics, ecological conditions and economic status of different regions, flexibly adjust the response strategy, and establish a scientific, systematic and comprehensive prevention and control system to curb the potential spread and harm of *Z. tau*. Currently, *Z. tau* has established stable populations in the southern regions of China (e.g., Guangxi, Guangdong, Guizhou, and Yunnan) and has caused significant economic losses to local fruit crops. The climatic conditions and cropping patterns in these regions provide ideal habitats and food sources. Monitoring and control strategies should be prioritised for areas where infestations have already occurred, including 1. The use of remote sensing technology and drone monitoring of orchards, combined with climate change modelling to predict areas of future suitability and the timely detection of new areas of expansion of *Z. tau*. 2. Enhance biological control practices and reduce the use of chemical pesticides by making use of natural enemies of pests, microbial agents, etc., to avoid long-term damage to the ecosystem. 3. Rational selection and use of low-toxicity, high-efficiency pesticides for targeted prevention and control. 4. Improve the agro-ecological environment and the self-regulatory capacity of the agricultural system by adjusting the crop layout and the ecological environment. 5. Reduce the chances of pest infestation by adjusting the structure of agricultural production and adopting crops of adaptable and pest-resistant varieties, and, at the same time, avoiding the cultivation of monoculture fruit trees at high densities. In addition, with the advancement of climate change, the potentially suitable area of *Z. tau* may be further extended to the central part of China. In order to address this potential threat, risk assessment and early warning systems need to be strengthened nationwide, and regionally differentiated prevention and control strategies need to be developed. These include: 1. Strengthening cooperation between different regions and establishing a cross-regional mechanism for prevention and control and a platform for information sharing, so as to ensure that prevention and control can be carried out in a synergistic manner across regions and sectors, and to minimise the waste of resources and improve the effectiveness of prevention and control. 2. Establish a long-term monitoring and evaluation mechanism to regularly check and evaluate the effectiveness of the implementation of control measures, and adjust the control strategy in a timely manner according to new climatic data and the expansion of the pest, so as to ensure that the control effect is long-lasting and effective. 3. Slowing down the proliferation of *Z. tau* through the setting up of isolation areas or buffer areas (e.g. isolating crop areas, orchards and wild habitats), and also strengthening ecological restoration and improving the local resilience of ecosystems, adopting diverse crop structures, and reduce food sources and habitat for *Z. tau*.

Although this study predicted the potential distribution range of *Z. tau* under different climate scenarios more accurately through ensemble modelling, certain limitations and uncertainties still exist. Firstly, the modelling in this study was mainly based on available environmental variables and climatic data, and failed to fully consider other biotic interactions (e.g. changes in natural enemies, host plants, etc.) that may affect the distribution of the species (54, 55). Second, the models are sensitive to the uncertainty of future climate change, and the prediction differences between different climate models may affect the accuracy of the results (56). In future studies, the complex relationship between climate change and species adaptation should be further explored, especially to reveal the physiological and ecological response mechanisms that *Z. tau* may exhibit under different climatic stresses. At the same time, species monitoring and management strategies should be optimized under dynamically changing climatic conditions, and a more prospective and flexible monitoring and early warning system should be established to effectively respond to the challenges posed by climate change. It is worth noting that future prevention and control strategies should not only focus on climate change factors, but also take into full consideration the impact of human activities on habitat pattern changes, so as to accurately predict the transmission dynamics of pests in a more realistic and integrated context, and develop scientific and effective prevention, control and management measures, which will support the sustainable development of agroecosystems.

## 5 Conclusion

In this study, we assessed for the first time the impacts of climate change and human activities on the potential geographic distribution pattern of *Z. tau* based on ensemble model and niche analysis. The results showed that *Z. tau* is mainly distributed in South and Central China. The addition of anthropogenic factors further expanded the range of suitable habitat for *Z. tau*, suggesting that human disturbance plays an important positive effect in its dispersal. Furthermore, climate warming may have contributed to the expansion of *Z. tau* into the central part of China by accelerating its reproduction rate, shortening its generation cycle, and improving its living environment. This result suggests that China will face a higher risk of transmission in the future and will need to develop appropriate control measures in advance. In addition, niche analyses indicated that the ecological stability of *Z. tau* remained moderate under different future climate scenarios, suggesting that it has strong environmental adaptation and ecological tolerance. In conclusion, this study not only reveals the spatial and temporal characteristics of the potential distribution of *Z. tau* driven by climate change and human activities, but also provides a scientific basis for future pest control and ecological management.

## Data Availability

The original contributions presented in the study are included in the article/supplementary material. Further inquiries can be directed to the corresponding authors.
